# Expression of Concern for Magnesium and Ketamine Gargle and Postoperative Sore Throat [Anesth Pain Med. 2015; 5 (3): e22367]

**DOI:** 10.5812/aapm-175518

**Published:** 2026-09-27

**Authors:** Mahmood Reza Alebouyeh

**Affiliations:** 1 Pain Research Center, Department of Anesthesiology and Pain Medicine, Iran University of Medical Sciences (IUMS) Tehran Iran

Dear Readers,

The editors and publisher of Anesthesiology and Pain Medicine journal wish to notify readers of a concern regarding one the articles published in 2015 (1).

Following receipt of a communication from an external whistleblower (Ticket #666540), drawing our attention to a potential issue that has been raised concerning aspects of this article. Concerns have been raised about the apparent similarity of error bars in Figure 2 and the percentages reported for male participants in Table 2. In particular, the reported values of 55% and 52.5% do not correspond to whole-person counts if each percentage was calculated from a group of 50 participants. These matters may affect the interpretation of the reported results.

The journal has requested an explanation and supporting data from the authors and is assessing the concerns. No conclusion about the reliability of the article or the cause of these discrepancies has been reached. This notice will be updated when the assessment is complete.

Sincerely Yours

Prof. Farnad Imani

Pain Research Center, Department of Anesthesiology and Pain Medicine, Iran University of Medical Sciences (IUMS), Tehran, Iran

Editor-in-Chief of Anesthesiology and Pain Medicine
